# First person – Pratibha Bhadauriya and Akanksha Onkar

**DOI:** 10.1242/bio.061923

**Published:** 2025-03-07

## Abstract

First Person is a series of interviews with the first authors of a selection of papers published in Biology Open, helping researchers promote themselves alongside their papers. Pratibha Bhadauriya and Akanksha Onkar are co-first authors on ‘ Glycogen synthase is required for heat shock-mediated autophagy induction in neuronal cells’, published in BiO. Pratibha conducted the research described in this article while a PhD student in Professor S. Ganesh's lab at the Indian Institute of Technology Kanpur, India. She is currently a project scientist in the same lab, focusing on understanding the molecular pathway behind stress-related disorders, including neurodegeneration and diabetes, by using cellular and mouse model systems. Akanksha is a postdoctoral research fellow in the lab of Dr Adrian Erlebacher at the University of California, USA, working at the intersection of glycoimmunolgy and pregnancy using mouse models and clinical samples, with an aim to improve maternal health.


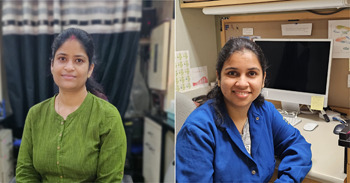


**Pratibha Bhadauriya and Akanksha Onkar**

**Describe your scientific journey and your current research focus**

**P.B.:** I began my scientific journey with an MTech degree from the National Institute of Technology, Allahabad, India, where I focused on characterising cold shock proteins. This early research experience provided a strong foundation for my transition into the study of stress-related disorders.

At Prof. Ganesh's lab, I further honed my skills in designing and conducting experiments, particularly those related to stress studies. My research explored the molecular pathways underlying neurodegenerative disorders, utilising cellular systems and complex mouse models to develop novel therapeutic strategies.

**A.O.:** I started my PhD in Prof. Ganesh's lab, where the focus was on uncovering the causes of neurodegenerative disorders. I was particularly drawn to a project investigating the role of glycogen synthesis in neuronal stress responses. The idea that something as fundamental as a sugar could aid neuronal survival fascinated me. For my thesis, I explored the role of glycogen in multiple stress conditions, including proteotoxic, oxidative, and heat shock stress using cellular, fly and mouse models. My findings established that glycogen and its synthetic machinery play a protective role in neurons under stress, reducing cytotoxicity and enhancing cell survival.

My passion for uncovering the diverse roles of sugars in various biological contexts led me to my current postdoc research, where I am investigating the role of glycans in maternal-fetal tolerance.

**Who or what inspired you to become a scientist?**

**P.B.:** My fascination with science began with a deep curiosity about the complexity of the human body and a desire to address health challenges. As a child, I was captivated by the intricate workings of life, from the smallest cells to the most complex structures. During my postgraduate studies, my PI nurtured this passion by introducing me to the transformative power of research and its direct impact on human health and well-being.

**A.O.:** The inspiration for my scientific career began during my master's program when I had the opportunity to complete a summer internship in a research lab. Witnessing the dedication of the scientists around me and their constant enthusiasm for discovery motivated me to pursue a path in research. The thrill of uncovering new insights and the potential impact of my research on improving human disease outcomes continue to drive my scientific journey.

**How would you explain the main finding of your paper**

Glycogen, a glucose polymer, serves as an energy reservoir in most major organs but remains scarce in neurons. This scarcity contrasts with the neuron's high energy demands and continuous need for glucose. Interestingly, under various stress conditions, neurons synthesize glycogen, yet the significance of this response remains largely unexplored. In this study, we investigated the role of glycogen and its synthesis machinery in neuronal heat stress and recovery. We found that glycogen levels increase during heat shock, a process requiring activation of the heat shock factor HSF1. Furthermore, we demonstrated that this glycogen accumulation, along with increased expression of its synthesizing enzyme, glycogen synthase, enhances autophagy – the cell's waste-clearing process – ultimately protecting neurons from heat stress-induced death.

**What are the potential implications of this finding for your field of research?**

This finding has significant implications for neuronal disease research, as it highlights a novel role for GS in regulating autophagy during heat stress via heat shock factor 1 (HSF1). Moreover, the study suggests that GS-mediated glycogen metabolism is not only reservoir of an energy but an active molecular player in heat stress responses. Targeting autophagy modulation in neurodegeneration and understanding the molecular mechanisms behind GS activation during heat shock could open new route for future research. Overall, this study provides a foundation for investigating GS as a potential therapeutic biomarker target for neuronal diseases, bridging the gap between metabolism, proteostasis, and neuronal survival under stress.

**Figure:**
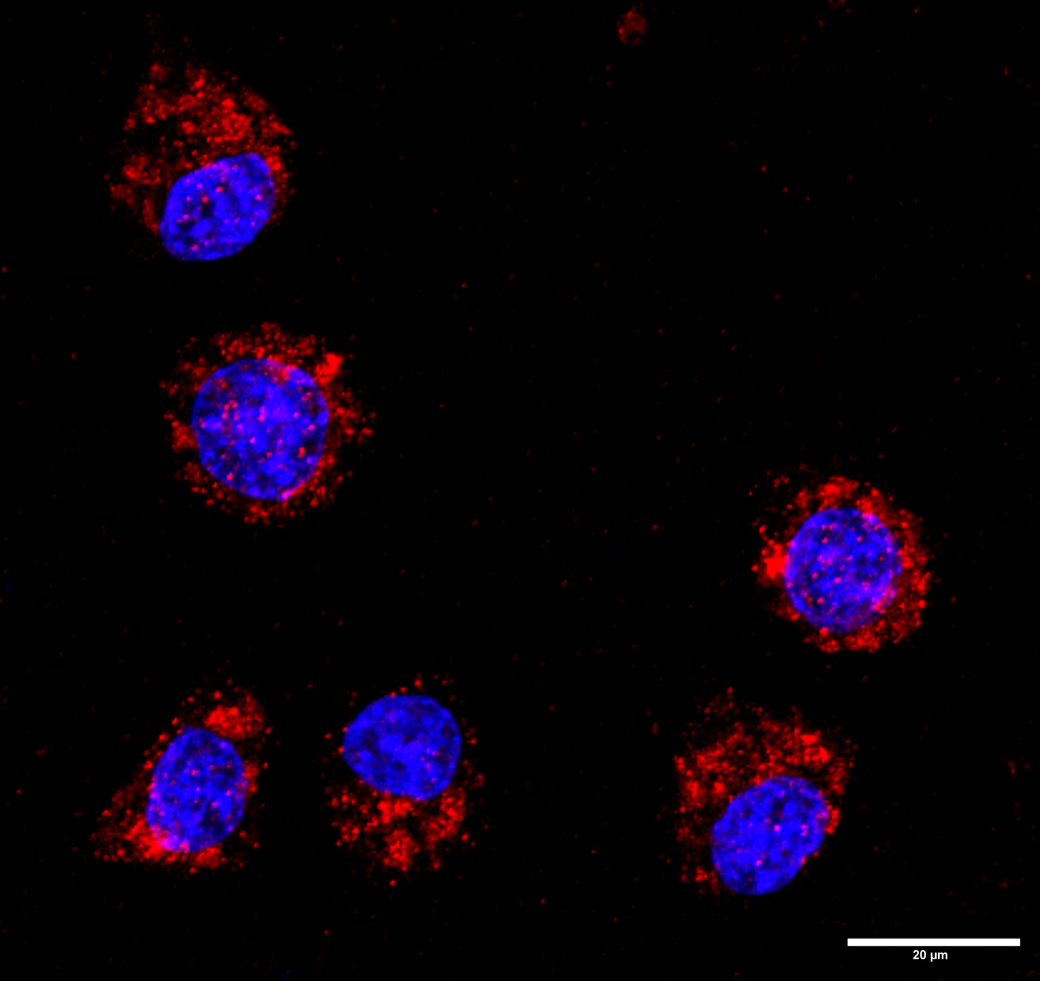
Confocal image showing the LC3 staining (red) in Neuro-2a cells during heat shock.

**Which part of this research project was the most rewarding?**

**P.B.:** The most rewarding part of this project was executing the hypothesis through well-coordinated, synergistic collaboration amongst all authors.

**A.O.:** The two most rewarding aspects of this project were, first, the discovery that heat stress induces a significant increase in glycogen levels – an unknown phenomenon before our study. Secondly, the project underscored the power of collaboration. It was a highly collaborative effort, with everyone contributing their expertise, and the discussions we had around experimental design and troubleshooting were incredibly fulfilling.

Above all, I am driven by the knowledge that my research has the potential to improve lives

**What do you enjoy most about being an early-career researcher?**

**P.B.:** As an early-career researcher, I deeply appreciate the thrill of discovery and the opportunity to make meaningful contributions to science. While the process can be challenging, it is equally rewarding – from formulating questions and building hypotheses to designing experiments and exploring new frontiers. Every success and setback are a valuable learning experience. Above all, I am driven by the knowledge that my research has the potential to improve lives, inspiring me to keep pushing boundaries and striving for new insights.

**A.O.:** For me, the most exciting part of being an early-career researcher is the thrill of discovery – knowing that you're generating new knowledge that no one else has seen before. Every experiment, every analysis, and every unexpected result has the potential to shift understanding in your field.

**What piece of advice would you give to the next generation of researchers?**

**P.B.:** My advice to the next generation of researchers is to foster a sense of wonder and determination in your scientific journey. Research often takes time, and the path is rarely linear, so cultivating patience and resilience is essential. Don't hesitate to think outside the box and ask bold questions. Embrace multidisciplinary learning and surround yourself with mentors and collaborators who inspire and support your growth.

**A.O.:** I have one key piece of advice for the next generation: stay curious and resilient. Research is full of challenges – failed experiments, rejected papers, and unexpected results – but every setback is an opportunity to learn. The key is not giving up and rising stronger each day. That's the essence of this field.

**What's next for you?**

**P.B.:** Moving forward, I am dedicated to deepening my understanding of neurodegenerative disorders by utilising cellular and mouse model systems to identify therapeutic targets. Additionally, I aim to enhance my contributions to the scientific community through mentorship, collaborations, and impactful publications.

**A.O.:** As the next step in my career, I plan to lead my own research group, where I will continue to explore the role of sugars in neurodegeneration, immune tolerance, and autoimmunity. Additionally, I aim to enhance my skills in the ever-evolving field of computational analysis, which I believe will be at the forefront of scientific research in the coming years.
